# DNase improves the efficacy of antimicrobial photodynamic therapy in the treatment of candidiasis induced with *Candida albicans*

**DOI:** 10.3389/fmicb.2023.1274201

**Published:** 2023-12-22

**Authors:** Cláudia Carolina Jordão, Marlise Inêz Klein, Paula Aboud Barbugli, Ewerton Garcia de Oliveira Mima, Tábata Viana de Sousa, Túlio Morandin Ferrisse, Ana Claudia Pavarina

**Affiliations:** ^1^Laboratory of Applied Microbiology, Department of Dental Materials and Prosthodontics, School of Dentistry, São Paulo State University (UNESP), Araraquara, Brazil; ^2^Department of Oral Diagnosis, Piracicaba Dental School, State University of Campinas (UNICAMP), Piracicaba, Brazil

**Keywords:** photochemotherapy, *Candida albicans*, antifungal drug resistance, fungi, enzyme

## Abstract

The study evaluated the association of DNase I enzyme with antimicrobial photodynamic therapy (aPDT) in the treatment of oral candidiasis in mice infected with fluconazole-susceptible (CaS) and -resistant (CaR) *Candida albicans* strains. Mice were inoculated with *C. albicans*, and after the infection had been established, the tongues were exposed to DNase for 5 min, followed by photosensitizer [Photodithazine^®^(PDZ)] and light (LED), either singly or combined. The treatments were performed for 5 consecutive days. Treatment efficacy was evaluated by assessing the tongues via fungal viable population, clinical evaluation, histopathological and fluorescence microscopy methods immediately after finishing treatments, and 7 days of follow-up. The combination of DNase with PDZ-aPDT reduced the fungal viability in mice tongues immediately after the treatments by around 4.26 and 2.89 log_10_ for CaS and CaR, respectively (versus animals only inoculated). In the fluorescence microscopy, the polysaccharides produced by *C. albicans* and fungal cells were less labeled in animals treated with the combination of DNase with PDZ-aPDT, similar to the healthy animals. After 7 days of the treatment, DNase associated with PDZ-aPDT maintained a lower count, but not as pronounced as immediately after the intervention. For both strains, mice treated with the combination of DNase with PDZ-aPDT showed remission of oral lesions and mild inflammatory infiltrate in both periods assessed, while animals treated only with PDZ-aPDT presented partial remission of oral lesions. DNase I enzyme improved the efficacy of photodynamic treatment.

## 1 Introduction

Oropharyngeal candidiasis (OPC) is the most prevalent infection caused by *Candida* spp. (Akpan and Morgan, 2002; Eggimman et al., 2003). Infections caused by *Candida* spp. are associated with biofilm formation, a complex microstructure of cells adhered to a surface and enveloped by an extracellular matrix (ECM) (Flemming et al., 2007). ECM contributes to preserving biofilms and conserving stable interactions between cells, surfaces (substrate), and the environment (Flemming et al., 2007). In addition, ECM reduces the susceptibility of microorganisms against therapies classically used (Flemming et al., 2007; Seneviratne et al., 2008). Biochemical analyses show that the production of polysaccharides (β-1-3-glucan, β-1-6-glucan, β-1-6-mannan and chitin, for example), nucleic acids (extracellular DNA—eDNA) and lipids protect the biofilm’ cells and keep stable interactions between ECM components (Eggimman et al., 2003; Nett et al., 2007; Martins et al., 2010). The antifungal resistance of *Candida albicans* biofilms is multifactorial, including the stimulation of drug efflux pumps, the physiological state of the cells, and the protection employed by the ECM through β-mannan and β-glucans that bind with fluconazole and amphotericin B (Flemming et al., 2007; Seneviratne et al., 2008). In addition to β-mannan and β-glucan, eDNA is an important constituent of the ECM and promotes the structural integrity of biofilms (Mitchell et al., 2016). The addition of DNase enzyme improves the susceptibility of mature *C. albicans* biofilms against some antifungal agents (Martins et al., 2010). Furthermore, the presence of polysaccharides or eDNA was reported as a bacterial biofilm mechanism of protection against the diffusion of antibiotics (Al-Fattani and Douglas, 2006; Anderson and O'Toole, 2008; Mulcahy et al., 2008).

Because the ECM has been related to biofilm protection (Nobile et al., 2008), the use of enzymes capable of hydrolyzing polysaccharides and nucleic acids has been investigated, as it represents an alternative way of increasing the susceptibility of the biofilm to antifungal drugs (Nobile et al., 2008). DNase I enzyme can significantly reduce eDNA, soluble matrix proteins, and water-soluble polysaccharides of a fluconazole-resistant *C. albicans* (Panariello et al., 2019). This enzyme acts externally to the cell, reducing biofilm stability and enhancing its susceptibility to photodynamic therapy and antifungals (Liao, 1974; Martins et al., 2012; Tetz and Tetz, 2016; Panariello et al., 2019). The treatment of mature biofilms with DNase I (50 mg/mL) inhibited adhesion, biofilm formation, and reduced the biomass by approximately 30% (Perezous et al., 2005). Incubation of *in vitro* 48 h-old biofilms for 5 min to DNase I reduced eDNA and extracellular polysaccharides in the ECM of fluconazole-susceptible and -resistant *C. albicans* strains (Panariello et al., 2019).

Due to the increase of resistant microorganisms (Kumar et al., 2022), the side effects of antifungals (Campoy and Adrio, 2017), recolonization, and organization into biofilms (Kumar et al., 2022), studies have evaluated alternative strategies to manage fungal infections. In this context, antimicrobial photodynamic therapy (aPDT) is suggested for inactivating microorganisms and treating oral candidiasis (Lambrechts et al., 2005; Konopka and Goslinski, 2007; Donnelly et al., 2008). The photodynamic process requires a photosensitizing agent (PS) combined with light with a wavelength corresponding to the PS absorption band (Donnelly et al., 2008). The interaction of light with PS, in the presence of oxygen, produces reactive species capable of inducing cell inactivation (Machado, 2000). Reactive species have non-specific reactivity with organic molecules and can cause irreversible damage to cellular targets, such as membrane lysis and protein inactivation. Thus, any cellular macromolecule can be considered a target for aPDT (Bonnett and Martínez, 2001; Donnelly et al., 2008).

Photodithazine (PDZ)-aPDT inactivated *Candida* biofilms and treated oral candidiasis (Seneviratne et al., 2008; Martins et al., 2010). A single application of PDZ-aPDT in a murine model decreased the fluconazole-susceptible *C. albicans* (ATCC 90028) viability by 4.36 log_10_ (Carmello et al., 2015). On the other hand, mice infected by a fluconazole-resistant *C. albicans* strain (ATCC 96901) that received a single session of PDZ-aPDT presented reduced fungal cell viability by 1.96 log_10_ (Alves et al., 2018). Five consecutive applications of PDZ-aPDT or antifungal nystatin promoted reductions in the fluconazole-susceptible *C. albicans* (ATCC 90028) by 3 and 3.2 logs_10_, respectively, and yielded the remission of tongue lesions after 24 h of treatment (Carmello et al., 2016). When the animals were inoculated with a fluconazole-resistant strain (ATCC 96901), PDZ-aPDT was as effective as the topical antifungal nystatin in the treatment, reducing viability by around 1.2 log_10_ (Hidalgo et al., 2019); however, the animals showed white or pseudomembranous patches on the dorsum of the tongues. In animals inoculated with fluconazole-resistant *C. albicans*, the associations of treatments (PDZ-aPDT and nystatin) reduced ~2.3 log_10_ of fluconazole-resistant *C. albicans* (Hidalgo et al., 2019), and the macroscopic analysis revealed remission of oral lesions ranging ~95% after 24 h (Hidalgo et al., 2019). In general, previous studies (Carmello et al., 2015, 2016; Alves et al., 2018; Hidalgo et al., 2019) demonstrated the efficacy of PDZ-aPDT in treating infections caused by fluconazole-susceptible *C. albicans*. However, fluconazole-resistant *C. albicans* have reduced susceptibility to aPDT, and to find similar outcomes in the treatment of infections with these strains, it is necessary to combine treatments.

DNase treatment might be an adjuvant to anti-biofilm therapies since it reduces most ECM components that can hinder antifungal drug penetration into biofilms without interfering with cell viability (Panariello et al., 2019; Abreu-Pereira et al., 2022). The incubation of fluconazole-susceptible *C. albicans* biofilm with DNase I (5 min) before PDZ-aPDT reduced the counting of viable colonies (CFU) and the quantity of eDNA in the ECM (Panariello et al., 2019). This treatment strategy applied to fluconazole-resistant *C. albicans* biofilm decreased CFU (~1.62 log_10_), water-soluble polysaccharides (36.3%), and eDNA (72.3%) (Abreu-Pereira et al., 2022). Hence, the effect of photodynamic treatment was potentiated because DNase I disturbed the ECM and allowed the diffusion of PDZ and light through the ECM of fluconazole-susceptible and -resistant *C. albicans* biofilm, increasing treatment efficacy (Abreu-Pereira et al., 2022). Hence, the present study evaluated whether the application of DNase could potentiate the action of PDZ-aPDT treatment in mice infected with fluconazole-susceptible and -resistant *C. albicans*, focusing on fungal viable population recovery and resolution of candidiasis lesions on the mice’s tongues.

## 2 Materials and methods

### 2.1 Photosensitizer, DNase enzime and LED parameters

Photodithazine^®^ (PDZ) is a chlorin e6 derivative (VETAGRAND, Co, Moscow, Russia), which has an absorption peak of 660 nm. The PDZ was prepared on the day of use from the stock solution (5,000 mg/L) at a concentration of 200 mg/L in natrosol gel (Farmácia Santa Paula, Araraquara, SP, Brazil) and was kept protected from light (Hidalgo et al., 2019). The bovine pancreas DNase I enzyme stock solution (AMPD1, Sigma-Aldrich, St. Louis, MO, USA) was prepared on the day of use in 0.1 M sodium acetate buffer (pH 5.5) at the concentration of 20 units/mL (Abreu-Pereira et al., 2022).

The red LED light device (LXHLPR09, Luxeon^®^ III Emitter, Lumileds Lighting, San Jose, California, USA) was used with an absorption band of 660 nm, and the light intensity at the end of the device (5 mm in diameter) was 44.6 mW/cm^2^. Thus, a light dose of 50J/cm^2^ (19 min) was applied to the tongues of animals infected with *C. albicans*.

### 2.2 Experimental oral candidiasis and treatments performed

The present study was approved by the Animal Ethics Committee of the School of Dentistry of Araraquara, UNESP (Case number: 09/2020). A total of 180 female mice of the Swiss strain (≅ 5 weeks old) were used from the vivarium of the School of Dentistry of Araraquara, UNESP. The animals were allocated in cages, with five animals per cage according to the study groups, and kept in a room with a controlled temperature (23 ± 2°C) with standard chow and water *ad libitum* (Carmello et al., 2016; Hidalgo et al., 2019).

Strains of *C. albicans* ATCC (American Type Culture Collection, Rockville, USA), susceptible to fluconazole (ATCC 90028; CaS) and resistant to fluconazole (ATCC 96901; CaR) were defrosted, reactivated on Agar Sabouraud Dextrose (SDA) medium (37°C, 48 h). Next, colonies were transferred and cultivated in RPMI 1640 (Sigma-Aldrich, St. Louis, MO, USA) buffered to a pH of 7.0 with 3-(N-Morpholino) propanesulfonic acid 4-Morpholinepropanesulfonic acid (MOPS) (Sigma-Aldrich, St. Louis, MO, USA) at 37°C for 16 h. Then, the cells were washed twice with sterile phosphate buffered saline (PBS) (50 mM), resuspended with 3 mL of RPMI 1640, and the cell suspension was standardized spectrophotometrically with an optical density (OD) of 540 nm (1.0 nm ± 0.08, corresponding to 10^7^ CFU/mL) (Carmello et al., 2016; Hidalgo et al., 2019).

**Figure 1:**
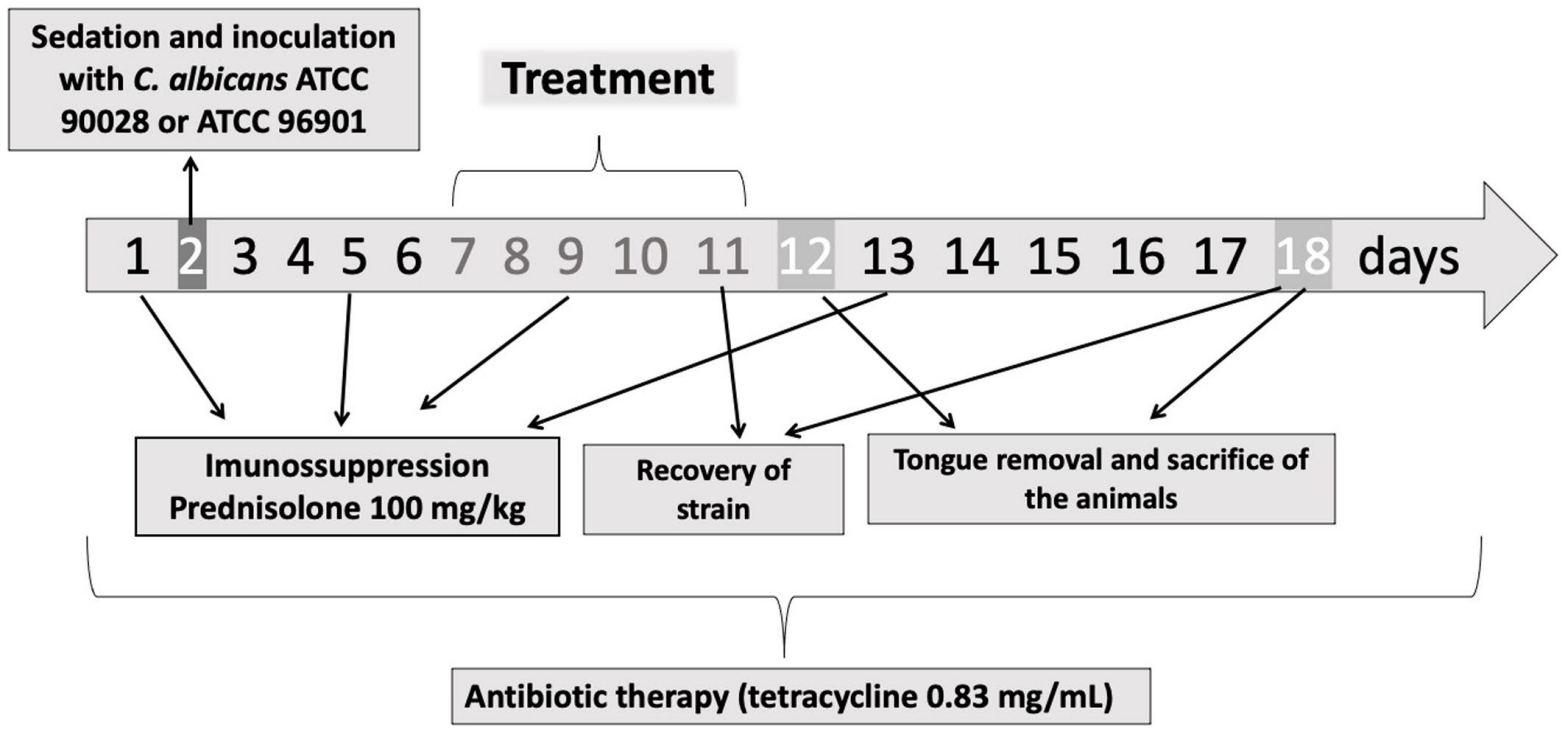
Experimental protocol. On the days 1, 5, 9, and 13 the animals were immunosuppressed with subcutaneous injections of prednisolone. On day 2, the animals were inoculated with CaS or CaR. The treatment was performed during 5 days (days 7–11). On days 11 and 18, the fungal load was recovered from the mice’s tongue. The tongue removal and euthanasia were performed on days 12 and 18. During the experimental period (18 days), tetracycline hydrochloride was administered in the water system.

For the induction of oral candidiasis, the methodology described before (Takakura et al., 2003; Carmello et al., 2016) was used with some modifications. Tetracycline (0.83 mg/mL) was administered in the water available to the animals during the experimental period. The animals were immunosuppressed with subcutaneous injections of prednisolone at a dose of 100 mg/kg of body mass on days 1, 5, 9, and 13. Inoculation with the strains was performed on day 2 of the experiment (Figure 1; Takakura et al., 2003; Carmello et al., 2016; Hidalgo et al., 2019). For this procedure, the animals were sedated with 0.1 mL of chlorpromazine hydrochloride (2 mg/mL), and sterile mini-swabs soaked in the CaS or CaR suspension were scrubbed across the dorsum of the animals’ tongues for 30 s.

On day 7, the presence of white patches or pseudomembranous lesions was verified, and the treatments were performed. The animals were anesthetized with an intraperitoneal injection of ketamine [100 mg/kg body weight (National Pharmaceutical Chemistry Union S/A, Embu, SP, Brazil)] and xylazine [10 mg/kg body weight (Veterinary JA Ltda., Sponsor Paulista, SP, Brazil)]. Then, the animals were placed in a supine position on the work table, the tongues were gently taken out of the oral cavity, and 50 μL of PDZ diluted in natrosol gel (200 mg/L) was applied with a pipette (Carmello et al., 2016; Hidalgo et al., 2019). The mice stayed in the dark for 20 min for a pre-incubation time. Then, each dorsum of the tongue was illuminated with LED for 19 min (50 J/cm^2^) (P + L+ group). The effect of the isolated application of PDZ (P + L-) and the LED (P-L+ group) was also evaluated. For DNase treatment, the tongue of the mice received 50 μL of DNase (20 units/mL) for 5 min. One group received the combined treatment with the enzyme and PDZ-aPDT (DNase+P + L+ group). After the treatments, neither DNase nor PDZ was removed from the mice’ tongue. The untreated control group (P-L- group) received no PDZ, light, or DNase. In addition, two additional negative infection control groups (NIC groups) with healthy animals were evaluated. In one of them, mice were immunosuppressed on days 1, 5, 9, and 13 (NIC+ group); in the other group, animals did not receive immunosuppression (NIC- group). Seven animals were evaluated for each experimental condition, except the group NIC+ (*n* = 3) and NIC- (*n* = 3). The therapies were performed once a day for five consecutive days (from day seven until day 11).

### 2.3 Fungal viable population

After five consecutive days of treatment (day 11) and 7 days after the end of the treatment (day 18), *C. albicans* cells were recovered from the tongue of mice. For this procedure, the mini-swabs were swabbed on the dorsum of each tongue for 1 min. Then, they were transferred to tubes with 1 mL of saline solution and vortexed for 1 min to detach *C. albicans* cells. Then, serial dilutions were made (10^−1^ a 10^−3^) and plated in duplicate in SDA culture medium containing 5 μg/mL of chloramphenicol. After 48 h of incubation at 37°C, the viable colonies were counted, and the values of CFU/mL were determined.

### 2.4 Macroscopic analysis of tongue’s lesions

The white patches or pseudomembranous lesions in the tongues of mice were photographed before the beginning of the treatments, 24 h (day 12), and 7 days (day 18) after the last application. All photographs were standardized and obtained with the same digital camera (Sony Cyber-Shot DSCF717; Sony Corporation, Tokyo, Japan), by the same operator and under the same conditions (place, light, angle, and position of the animals), thus aiming to facilitate the reproducibility. The extension area of the lesion on the tongue in each photograph was evaluated using the ImageJ.exe program.^1^ The percentage of the extension area of each lesion over the total area of the tongue was calculated using this software (Hidalgo et al., 2019).

### 2.5 Histopathological analyses and animal sacrifice

Initially, mice were anesthetized with an intraperitoneal injection of ketamine and xylazine. Then, animals’ tongues were surgically removed and destinated for histopathological and fluorescence microscopic analyses. After the tongue excision, mice were euthanized by intramuscular injection of a lethal dose of ketamine (0.2 mL) and xylazine (0.4 mL) 24 h (day 12) and 7 days (day 18) after the last application of treatment (Carmello et al., 2016; Hidalgo et al., 2019).

The tongues were placed in plastic cassettes for the histopathological and fluorescence microscopic analyses. These cassettes were immersed in 10% paraformaldehyde (pH 7.2) (441.244, Sigma-Aldrich, St Louis, MO, USA). Then, the histological fixation process was made, and the blocks were fixed on wooden supports and placed in a rotating microtome. Sixteen serial histological sections of each block were obtained. These cuts were placed on glass slides and stained with hematoxylin–eosin (HE) stain to evaluate the histological events that occurred in each of the groups through light microscopy [Zeiss microscope LSM 700 (Carl Zeiss, Heidelberg, Germany)] at 100 and 200X magnification. A pathologist performed the histological analysis, and the following aspects were evaluated: the presence/absence of yeast and inflammatory infiltrate, epithelial tissue integrity, and adjacent connective tissue response. The material was classified into scores: 0—the absence of inflammation; 1—the presence of inflammatory infiltrate; 2—moderate inflammation; 3: severe inflammation; and 4: abscess formation (ISO 7405:1997). The evaluation was performed by a single examiner blinded to each experimental group at each evaluated time after treatment.

### 2.6 Microscopy analysis of fluorescence to determine fungal colonization on tongues

Initially, the samples were deparaffinated and hydrated in water. The antigenic retrieval was performed by heat. The sections were then immersed in 10 mM buffered sodium citrate, pH 6.0, and placed in the microwave twice for 5 min each (El-Habashi et al., 1995). Next, the slides were dried, and the sections were circled with a hydrophobic barrier pen (Sigma Advanced PAP Pen-Z377821) and 20 μL of the primary antibody (1 → 4)-β-mannan and galacto-(1 → 4)-β-mannan (400–4) (Table 1) diluted in 2% bovine serum albumin (BSA) and 0.1% Triton X100 (1:20 dilution) was pipetted on each section (Lobo et al., 2019). The slides were incubated overnight (4°C). After incubation, sections were carefully washed with 0.89% NaCl solution, and a blocking solution (3% BSA) was added, followed by incubation for 15 min (room temperature). Then, the sections were washed again with 0.89% NaCl solution, and the secondary antibody (20 μL) labeled with Alexa Fluor® 594 nm (1:500 dilution in 2% BSA) was added (Table 1), followed by incubation for 2 h (4°C). After the secondary antibody incubation time, the sections were washed with 0.89% NaCl and incubated with 20 μL of concavalin-A lectin conjugated with Alexa Fluor^®^ 488 nm (200 μg/mL) (Table 1) and Hoescht (6 μg/mL) (Table 1) for 30 min. Next, samples were washed with 0.89% NaCl. The mounting media [Fluoromount ^™^ Aqueous Mounting Medium (F4680, Sigma-Aldrich, St Louis, MO, USA)] was added, and the slides were ready for image acquisition. Images were acquired using the Leica DM2500 LED microscope (Leica Microsystems, Wetzlar, Germany).

**Table 1:** Probes and stains used in the fluorescence microscopic analysis.

Labeling target	Labeling (stain or probe) (excitation/emission nm)	Brand	References
*C. albicans*	ConcanavalinA (ConA) lectin conjugated with tetramethylrhodamine (488/520 nm)	Molecular Probes (Cat. No. C860)	Lobo et al. (2019) and Falsetta et al. (2014)
Nucleic acids	HOESCHT 33342	ABCAM Staining Staining Dye Solution (Cat. No. ab228551)	Chazotte (2011)
Polysaccharides produced by *C. albicans*	Primary monoclonal antibody to (1 → 4)-β-mannan and galacto-(1 → 4)-β-mannan	Biosupplies (Cat. No. 400-4)	Lobo et al. (2019) and Wartenberg et al. (2014)
Secondary antibody	Goat Anti-Mouse IgG H&L (Alexa Fluor^®^ 594) (561/620 nm)	Abcam (Cat. No. ab175660)	Lobo et al. (2019) and Wartenberg et al. (2014)

### 2.7 Statistical analysis

Analyses were performed using the IBM SPSS Statistics for Windows Version 27; IBM Corp., Armonk, NY, USA. Data from each strain was evaluated separately. The normality and homoscedasticity of the data from CFU converted in base-10 logarithms for each strain were assessed using the Shapiro–Wilk and Levene’s tests, respectively. The data were normal and heteroscedastic, so they were analyzed by a two-way ANOVA test, considering two treatment evaluation periods (immediate and 7 days after). Games-Howell post-hoc analysis was performed for multiple comparisons (*α* = 5%). The percentage values of tongue’s lesions assumed normality and were homoscedastic for the data evaluated in both periods (24 h and 7 days after the treatments) for CaS and CaR. Thus, they were analyzed by one-way ANOVA, followed by Tukey’s post-hoc (*α* = 5%). Descriptive analyses were performed for the images obtained for the histopathological and fluorescence microscopy evaluations.

## 3 Results

### 3.1 Fungal viable population from mice inoculated with CaS and CaR

**Figure 2:**
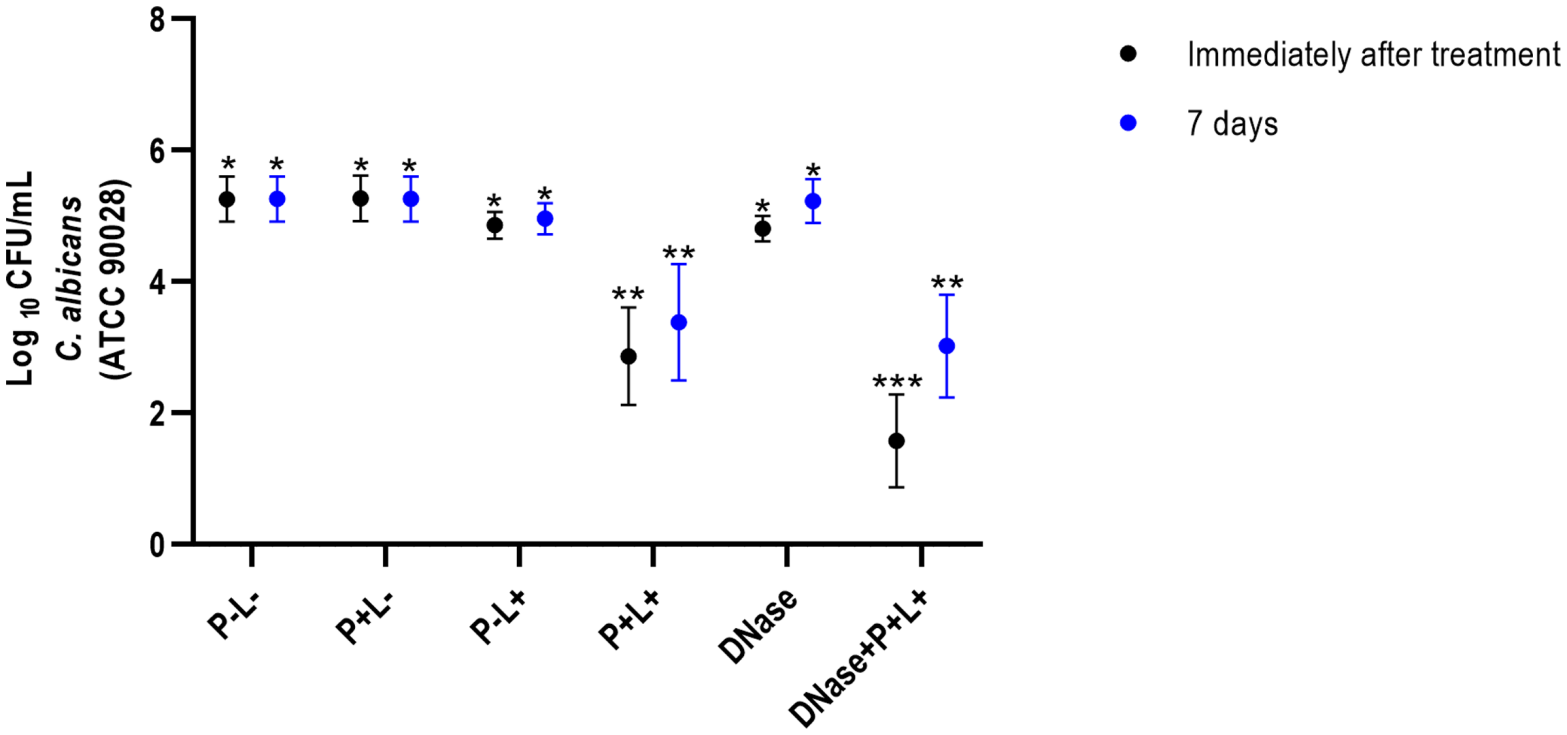
Mean values +/− standard deviation of log_10_ (CFU/mL) for different experimental groups and periods evaluated (immediately and 7 days after treatments) for animals inoculated with CaS. Different number of asterisks denotes statistical difference between the groups.

The results of viability from CaS immediately after the treatments demonstrated that the animals treated with DNase followed by PDZ-aPDT (DNase+P + L+ group) showed the highest log_10_ reduction value (CFU/mL), compared to the negative control group (P-L-) equivalent to 4.26 log_10_ (Figure 2) and different from the other groups and the control (*p* ≤ 0.0001). The P + L+ group (PDZ-aPDT) showed a reduction of approximately 2.50 log_10_ compared to the control (P-L- group) (Figure 2). The other groups showed statistically similar values with the control (P-L-) (*p* ≥ 0.05) (Figure 2).

**Figure 3:**
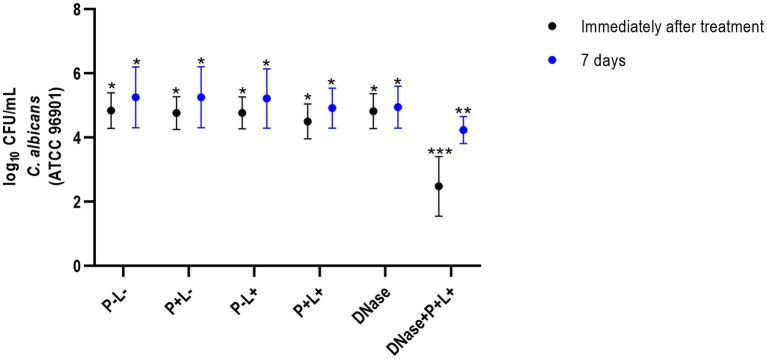
Mean values +/− standard deviation of log_10_ (CFU/mL) for different experimental groups and periods evaluated (immediately and 7 days after treatments) for animals inoculated with CaR. Different number of asterisks denotes statistical difference between the groups.

The results of CaR showed that DNase+P + L+ group immediately after the treatments was statistically different from the other groups and exhibited the highest log_10_ reduction value (CFU/mL), compared to the P-L-group (*p* ≤ 0.0001), equivalent to 2.89 log_10_ (Figure 3). The group treated only with PDZ-aPDT (P + L+ group) showed a reduction of 0.34 log_10_ compared to the P-L- group. The P + L-, P-L+, P + L+, DNase and P-L-groups presented statistically similar effects (Figure 3).

The results of 7 days after the end of the treatments demonstrated that the DNase+P + L+ group exhibited the greatest reduction in the viable colonies of CaS when compared to the negative control group (P-L-) (*p* ≤ 0.0001); this reduction was approximately 1.97 log_10_ (Figure 2). The P + L+ group was similar to DNase+P + L+ group and showed a statistically different value from the other groups, with a reduction of 1.18 log_10_ compared to the P-L- group (*p* ≤ 0.0001). The other experimental groups (P + L-, P-L+, DNase) showed statistically similar effects among themselves and with the negative control (P-L-) (*p* ≥ 0.05) (Figure 3).

For CaR after 7 days of the end of the treatments, the DNase+P + L+ group showed the greatest reduction in viable colonies when compared to the P-L- group (*p* ≤ 0.0001), with a reduction of approximately 1.27 log_10_ (Figure 3). The P + L+ group showed a reduction of 0.22 log_10_ compared to the P-L- group. The other experimental groups (P + L-, P-L+, P + L+, and DNase) showed statistically similar values among themselves and the P-L- group (*p* ≥ 0.05) (Figure 3).

### 3.2 Clinical evaluation from mice inoculated with CaS and CaR

**Figure 4:**
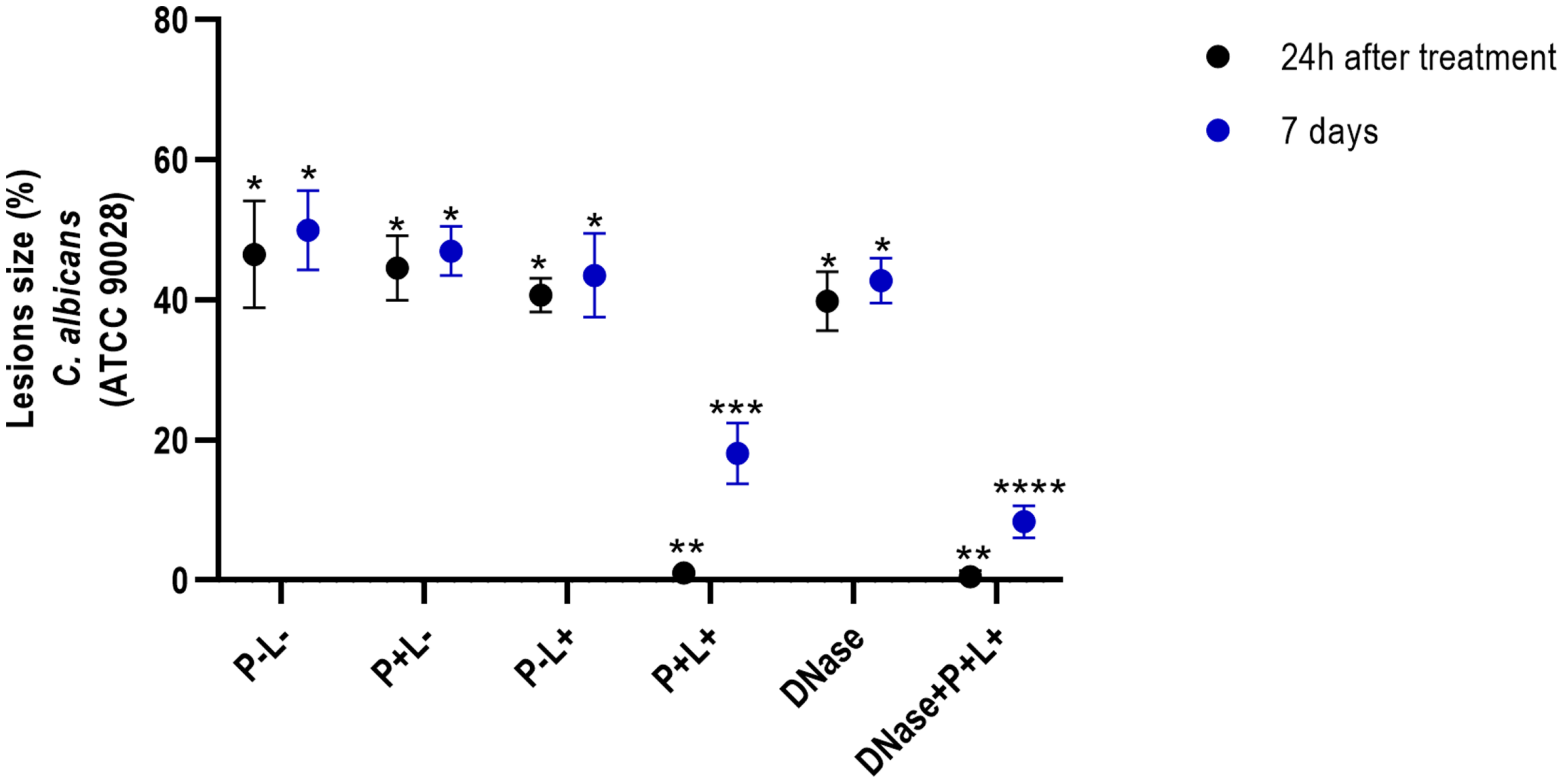
Mean values +/− standard deviation of the lesion size (with the size of the patches) in percentages (%) on the tongue’s dorsum of the mice inoculated with CaS evaluated 24 h (black circle) and 7 days (blue circle) after the end of the treatments. * denotes statistical difference. Different number of asterisks denotes statistical difference between the groups.

**Figure 5:**
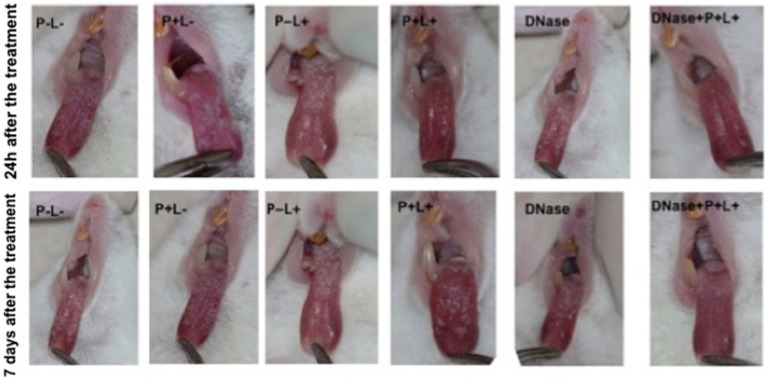
Representative images of the white or pseudomembranous patches of mice’ tongues inoculated with CaS for the groups P-L-, P + L-, P-L+, and DNase 24 h and 7 days after the end of treatment. Also, representative images of the remission of tongue lesions were observed in the mice submitted to the P + L+ 24 h and DNase+P + L+ 24 h and 7 days after the treatments.

The results after 24 h of treatment (Figure 4) for mice inoculated with the CaS showed that the DNase+P + L+ and P + L+ groups significantly reduced oral lesions by 98.92 and 97.71%, respectively, when compared to the P-L- group (*p* ≤ 0.0001). The results for 7 days after the treatments showed a reduction in oral lesions of 83.31% for DNase+P + L+ group, compared to the P-L- group (*p* ≤ 0.0001). The DNase+P + L+ group was statistically different from P + L+ group that reduced the oral lesions by around 63.81% compared to the P-L- group (*p* ≤ 0.0001). The other groups evaluated immediately and after 7 days (Figure 4) showed a statistically similar effect to the P-L- control (*p* ≥ 0.05). The images presented in Figure 5 illustrate the presence of white patches or pseudomembranous plaques on the dorsum of the animals’ tongues inoculated with CaS for each group 24 h and 7 days after the treatments.

**Figure 6:**
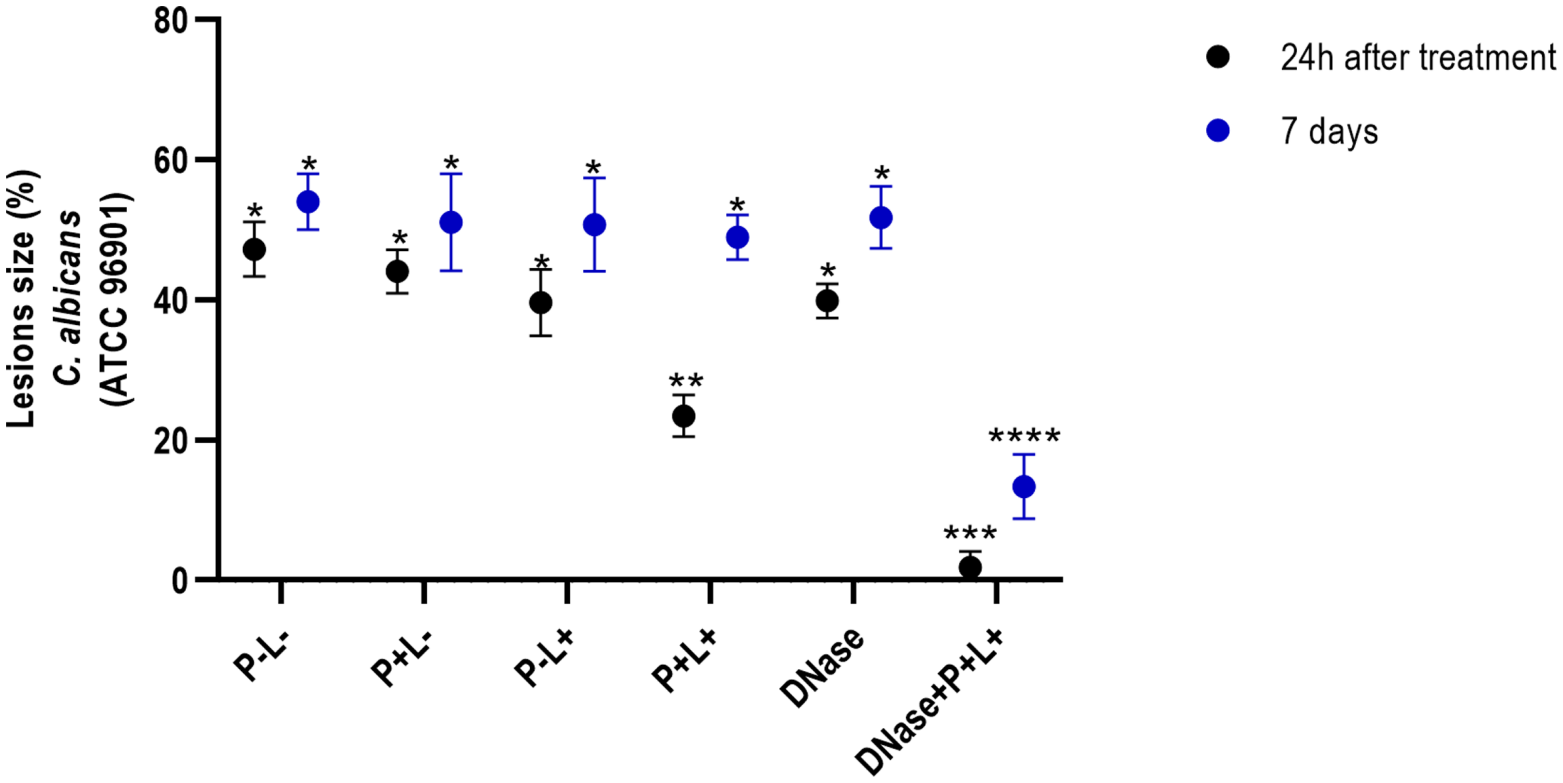
Mean values +/− standard deviation of the lesion size (with the size of the patches) in percentages (%) on the tongue’s dorsum of the mice inoculated with CaR evaluated 24 h (black circle) and 7 days (blue circle) after the end of the treatment. * denotes statistical difference. Different number of asterisks denotes statistical difference between the groups.

The results obtained for CaR (Figure 6) demonstrated that the DNase+P + L+ and P + L+ groups immediately after the treatments exhibited reductions of oral lesions by 96.07 and 50.41%, respectively, when compared to the P-L- group (*p* ≤ 0.0001). Immediately after the treatments, the other groups showed a statistically similar effect to the P-L- group (p ≥ 0.05). In addition, 7 days after the treatments, the DNase+P + L+ group showed a significant reduction in oral lesions by 75.24% when compared to the P-L- group (*p* ≤ 0.0001). The other groups showed statistically similar values to the P-L- control (*p* ≥ 0.05) (Figure 6).

**Figure 7:**
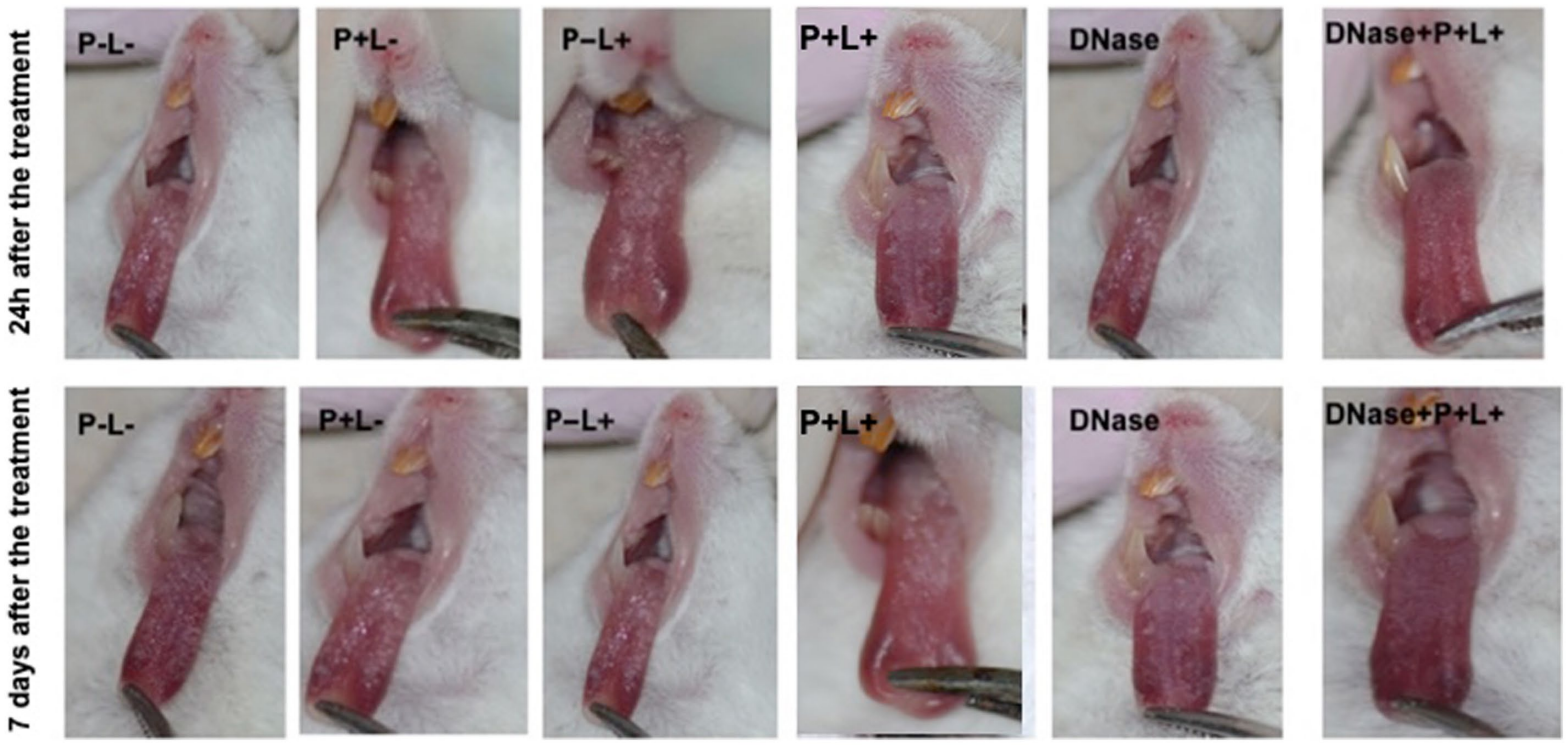
Representative images of the white or pseudomembranous patches of mice’ tongues inoculated with CaR for the groups P-L-, P + L-, P-L+, P + L+, and DNase 24 h and 7 days after the end of treatment. Also, representative images of the remission of tongue lesions in the mice submitted to the DNase+P + L+ treatment are observed 24 h and 7 days after the treatment.

The images in Figure 7 illustrate the presence of white patches or pseudomembranous plaques on the dorsum of the tongues 24 h and 7 days after the treatments performed on animals inoculated with CaR (Figure 7).

### 3.3 Histopathological evaluation

**Figure 8:**
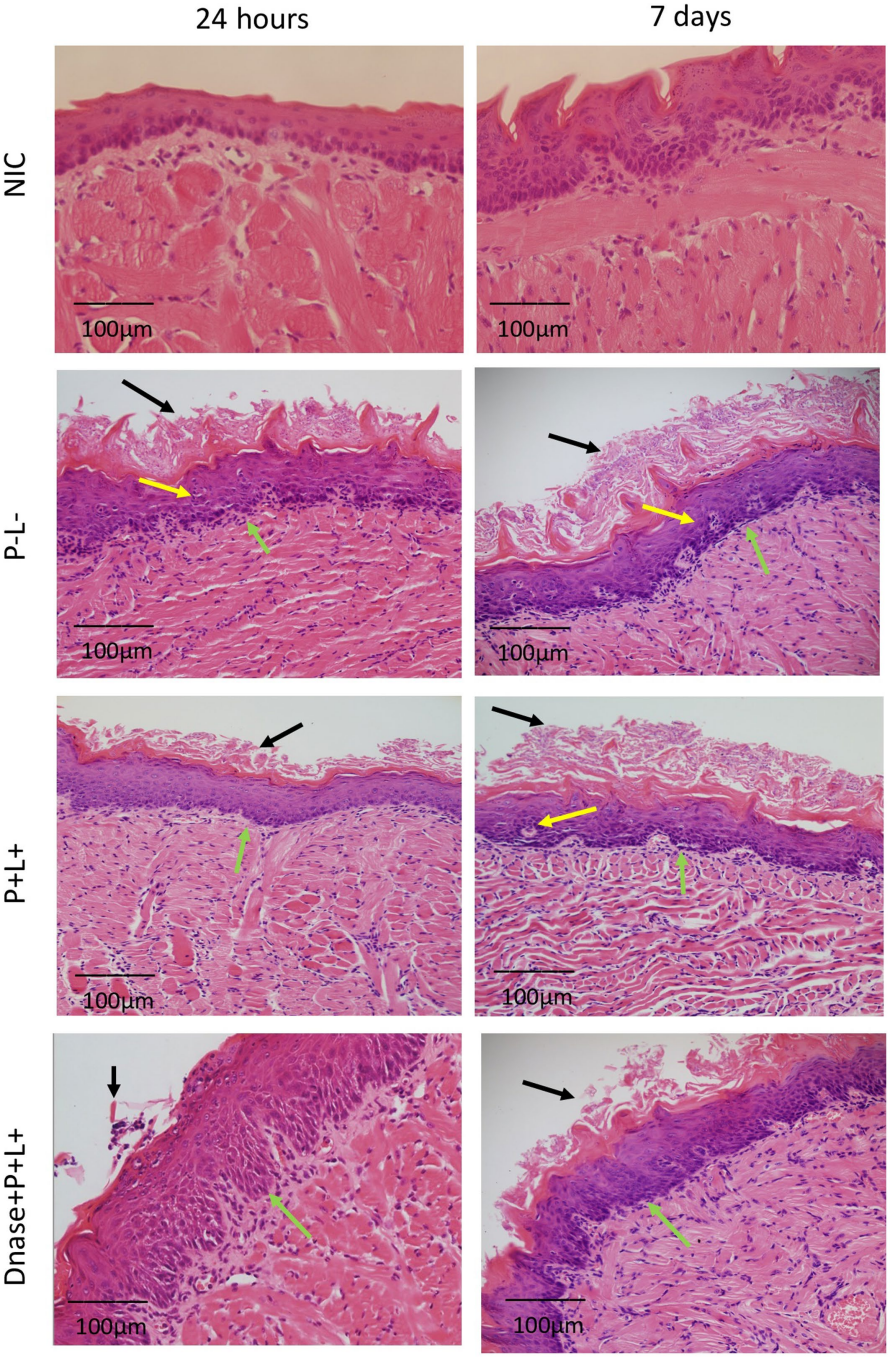
Representative images of the histological sections of tongues from mice inoculated with CaS and recovered 24 h and 7 days after the end of treatments. Muscle tissue was stained with hematoxylin–eosin (HE) (40X). Black arrow – keratin layer contaminated with hyphae and pseudohyphae; green arrow – lamina propria and yellow arrow – dilated blood vessels in response to the local inflammatory reaction.

The histopathological evaluation demonstrated that the sections from tongues contaminated with CaS exhibited mild inflammatory infiltrates for groups DNase+P + L+ and P + L+ 24 h after the treatment (Figure 8). These groups presented histopathological characteristics similar to those observed in the NIC- and NIC+ groups. The stratified epithelium exhibited normal and healthy features, with lingual papillae covered by a fine keratin layer. The other groups (P-L-, P-L+, P + L- and DNase) presented similar histopathological characteristics with moderate inflammatory infiltration and the presence of numerous hyphae/pseudohyphae on the keratin layer and some hyphae/pseudohyphae invading the epithelial tissue of the tongues (Figure 8). Regarding the histological sections evaluated at 7 days after the treatment for CaS, the morphological characteristics remained relatively unchanged, with the exception of the P-L-, P + L-, P-L+, and DNase groups, which showed moderately degraded muscle tissue (Figure 8).

**Figure 9:**
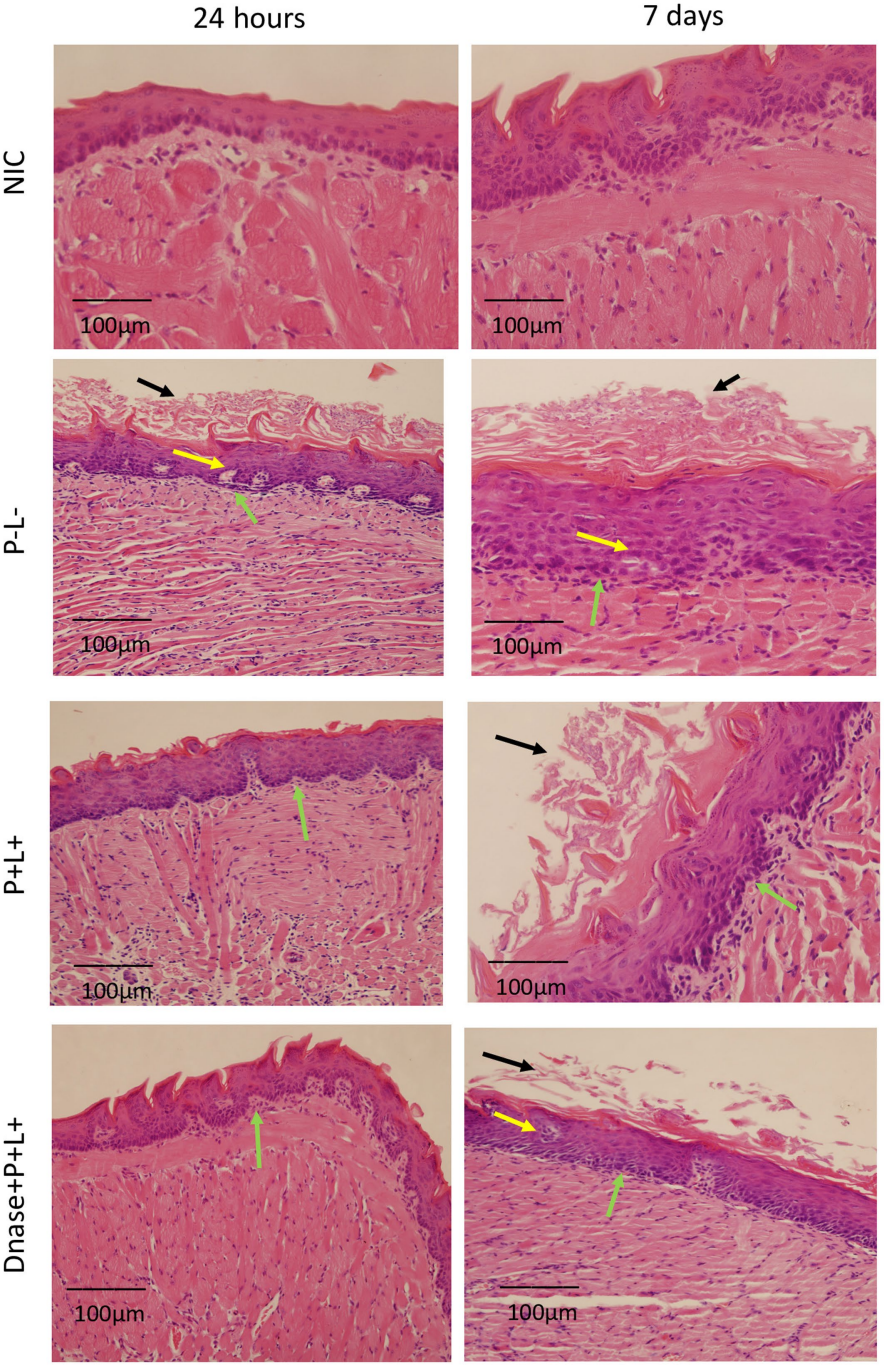
Representative images of the histological sections of tongues from mice inoculated with CaR and recovered 24 h and 7 days after the end of treatments. Muscle tissue was stained with hematoxylin–eosin (HE) (40X). Black arrow – keratin layer contaminated with hyphae and pseudohyphae; green arrow – lamina propria and yellow arrow – dilated blood vessels in response to local inflammatory reaction.

For the animals inoculated with CaR (Figure 9), the group treated with DNase+P + L+ presented histopathological characteristics similar to those observed in the NIC- and NIC+ groups (Figure 8) after 24 h of the treatments. The stratified epithelium exhibited normal and healthy features, with lingual papillae covered by a fine keratin layer (Figure 9). The group treated with P + L+ showed a greater number of hyphae and pseudohyphae within the keratin epithelial layer. The animals in the P-L-, P + L-, P-L+, and DNase groups presented extensive amounts of *C. albicans* covering the epithelial tissue, and there was a loss of the papillae (Figure 9). This epithelium showed intense inflammation with the existence of mononuclear cells inside the dilated blood vessels caused by the inflammation. The underlying connective tissue was formed by muscle fibers with normal characteristics.

The histological findings 7 days after treatments (Figure 9) for the P-L-, P + L-, P-L+, and DNase groups contaminated with CaR were similar to those observed 24 h after treatments associated with damage in the muscular tissue. In the P + L+ and DNase+P + L+ groups, many hyphae and pseudohyphae were observed in the epithelium keratin layer, but the epithelial tissue remained with normal characteristics (Figure 9).

### 3.4 Microscopy fluorescence evaluation

**Figure 10:**
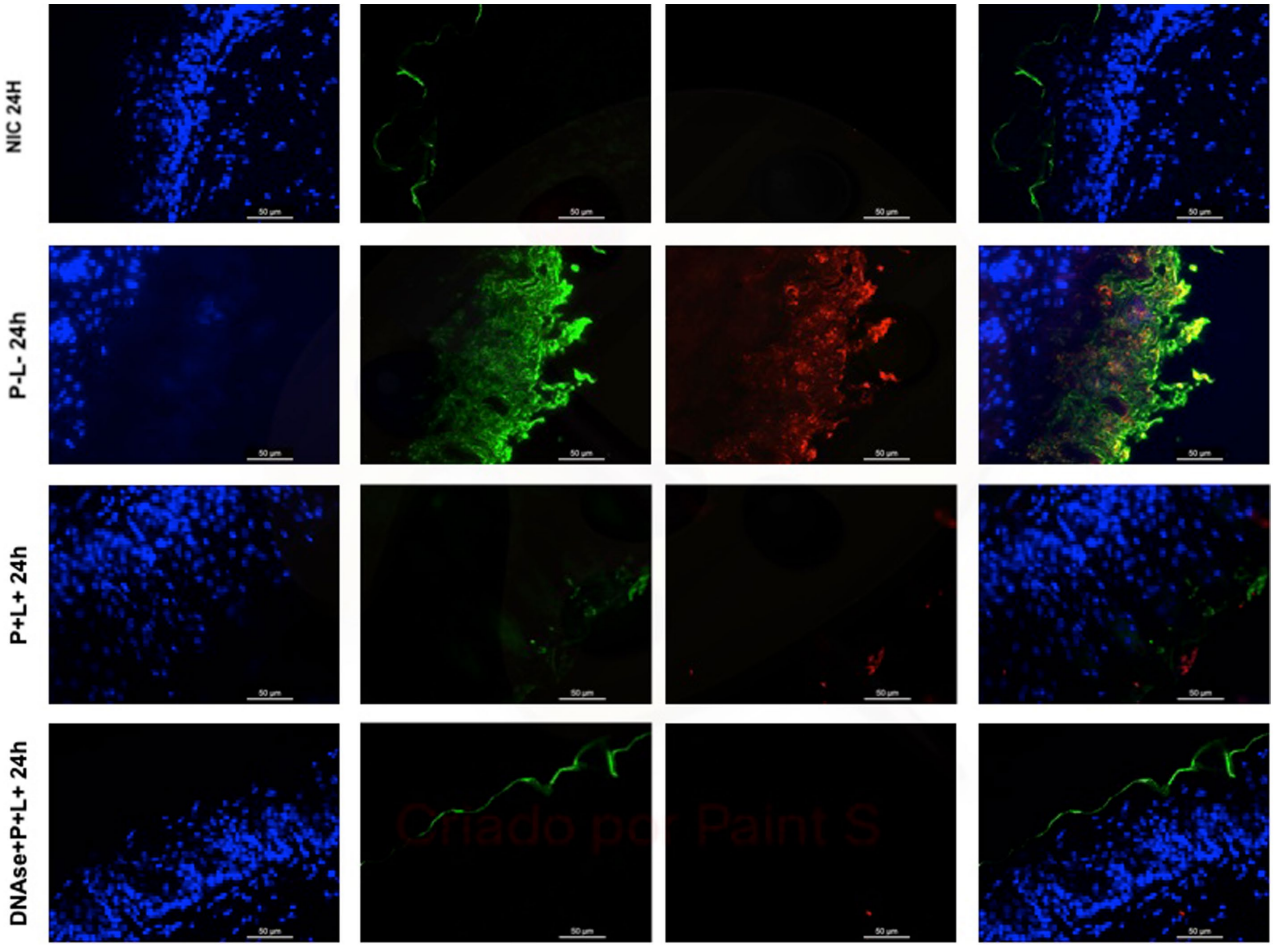
Representative fluorescence microscopy images of histological tongues sections recovered 24 h after treatments from the mice inoculated with CaS, labeled with Hoestch (first left column), concanavalin A conjugated with Alexa 488 nm (second column), and with primary antibody (1 → 4)-β-mannan and galacto-(1 → 4)-β-mannan (400–4) paired with secondary antibody conjugated with Alexa Fluoride 594 nm (third column). The tongue tissue cells (cell nucleus) were represented by the color blue. Fungal cells and matrix polysaccharides of *C. albicans* were represented by the colors green, and red, respectively. In the last column are the merged images.

**Figure 11:**
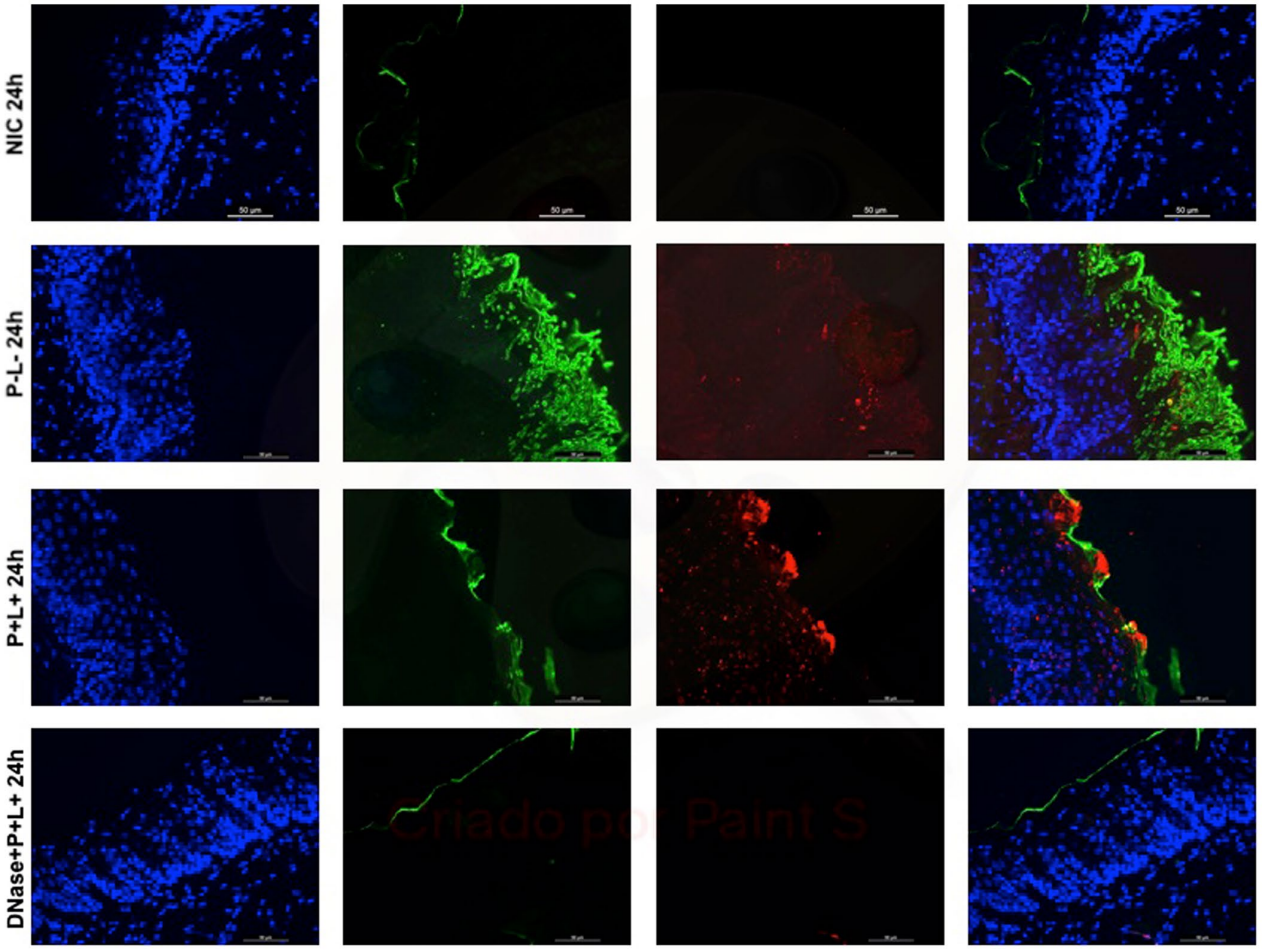
Representative fluorescence microscopy images of histological tongues sections recovery 24 h after the treatments from the mice inoculated with CaR, labeled with Hoestch (first left column), concanavalin A conjugated with Alexa 488 nm (second column), and with primary antibody (1 → 4)-β-mannan and galacto-(1 → 4)-β-mannan (400–4) paired with secondary antibody conjugated with Alexa Fluoride 594 nm (third column). The tongue tissue cells (cell nucleus) were represented by the color blue. Fungal cells and matrix polysaccharides of *C. albicans* were represented by the colors green and red, respectively. In the last column are the merged images.

The representative images from animals for P-L- group inoculated with CaS and CaR 24 h after the treatment (Figures 10, 11, respectively) showed a thick layer of biofilm (green) surrounded by polysaccharides (1–4 β-mannan and galacto) (red) produced by *C. albicans*. The images of DNase+P + L+ group presented similarity with NIC group once the polysaccharides (red) and fungal cells (green) were not labeled. The P + L+ group showed a small amount of biofilm (green) and polysaccharides (red) (Figures 10, 11).

**Figure 12:**
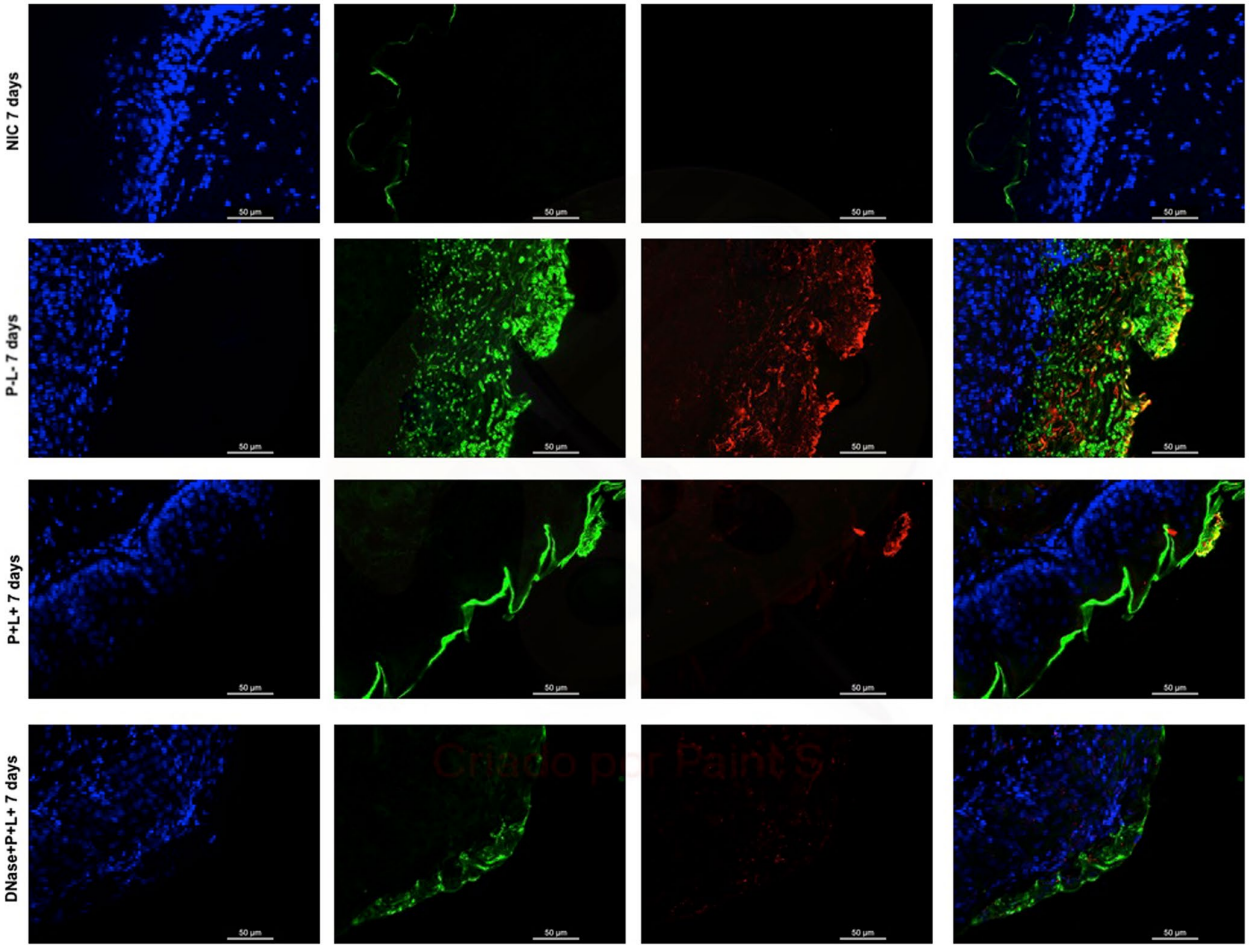
Representative fluorescence microscopy images of histological tongues sections recovered 7 days after the end of treatments from the mice inoculated with CaS, labeled with Hoestch (first left column), concanavalin A conjugated with Alexa 488 nm (second column), and with primary antibody (1 → 4)-β-mannan and galacto-(1 → 4)-β-mannan (400–4) paired with secondary antibody conjugated with Alexa Fluoride 594 nm (third column). The tongue tissue cells (cell nucleus) were represented by the color blue. Fungal cells and matrix polysaccharides of *C. albicans* were represented by the colors green and red, respectively. In the last column are the merged images.

**Figure 13:**
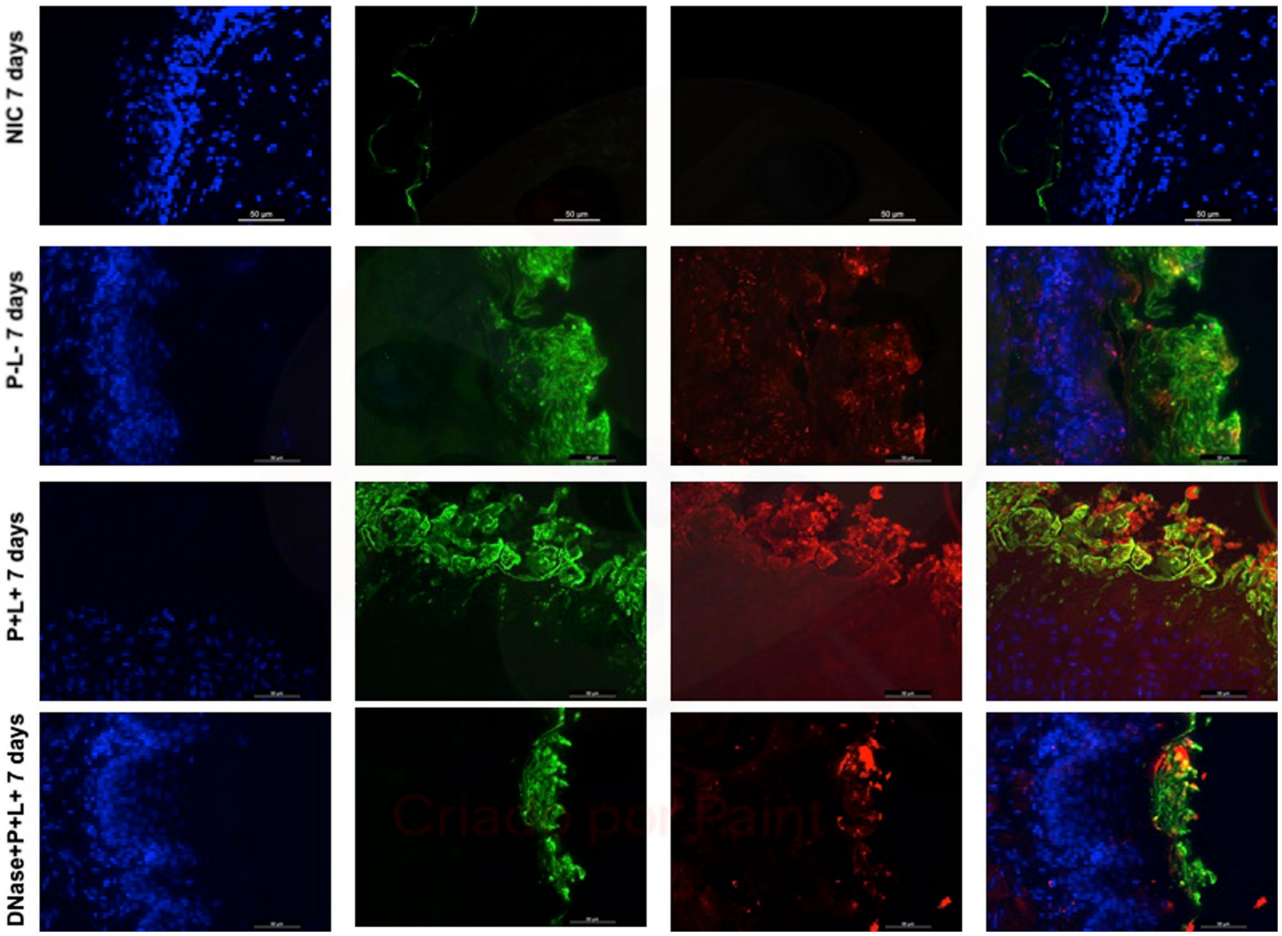
Representative fluorescence microscopy images of histological tongues sections recovery 7 days after the end of treatments from the mice inoculated with CaR, labeled with Hoestch (first left column), concanavalin A conjugated with Alexa 488 nm (second column), and with primary antibody (1 → 4)-β-mannan and galacto-(1 → 4)-β-mannan (400–4) paired with secondary antibody conjugated with Alexa Fluoride 594 nm (third column). The tongue tissue cells (cell nucleus) were represented by the color blue. Fungal cells and matrix polysaccharides of *C. albicans* were represented by green and red, respectively. In the last column are the merged images.

After 7 days of the treatment for CaS and CaR (Figures 12, 13), the P-L- group was labeled with polysaccharides produced by *C. albicans* (red), with higher intensity of label in fungal cells (green) (Figures 12, 13). The DNase+P + L+ group of animals contaminated with CaS presented a small layer of *C. albicans* biofilm and polysaccharides (red color) (Figure 12). The P + L+ group demonstrated a slight presence of fungal biofilm (green) and polysaccharides (red color) (Figure 12). For CaR, the group DNase+P + L+ presented less biofilm and polysaccharides than the group treated only with PDZ-aPDT (P + L+ group) once there was a thick layer of CaR biofilm (green) surrounded by polysaccharides (red) (Figure 13).

## 4 Discussion

*In vitro* studies report the improved efficacy of PDZ-aPDT combined with DNase against *C. albicans* biofilms (Panariello et al., 2019; Abreu-Pereira et al., 2022). Enzymes decrease the integrity of the ECM by hydrolyzing proteins, polysaccharides, and nucleic acids. DNase I reduces biofilm stability, increasing the susceptibility of the biofilm to the action of antifungals and aPDT (Liao, 1974; Martins et al., 2012; Tetz and Tetz, 2016; Panariello et al., 2019; Abreu-Pereira et al., 2022). The current study investigated whether applying DNase I enzyme could potentiate PDZ-aPDT outcomes in mice infected by susceptible- and fluconazole-resistant *C. albicans*. Here, DNase I (20 units/mL) combined with PDZ-aPDT promoted antifungal effects against CaR in the oral lesions of mice with experimental oral candidiasis, and macroscopic analysis showed that 24 h after completion of treatment, the animals presented 96.7% remission of lesions. To our knowledge, no previous published study has investigated *in vivo* the efficacy of DNase I associated with aPDT in treating induced oral candidiasis in mice.

The results from mice inoculated with CaR showed that DNase I prior PDZ-aPDT promoted a reduction in viable colony counts by around 2.89 and 1.27 log_10_, respectively, immediately and 7 days after the treatments. In addition, the macroscopic oral lesions were reduced by around 96.07 and 75.24% 24 h and 7 days after the treatment, respectively. These results observed here corroborate with a previous study (Hidalgo et al., 2019), that evaluated the combination of PDZ (200 mg/L) mediated aPDT associated with nystatin in the treatment of mice infected with fluconazole-resistant *C. albicans* (ATCC 96901). Using the same methodology for induction of infection in mice, the combination of treatments reduced ~2.60 log_10_ the fungal viability and ~ 95% the macroscopic oral lesions 24 h after the treatment (Hidalgo et al., 2019). The results obtained after 7 days of the end treatment showed that nystatin combined with PDZ-aPDT promoted a reduction of ~1 log_10_ in fungal viability and macroscopic reduction in oral lesions by around ~50% (Hidalgo et al., 2019). Here, DNase yielded an outcome more favorable than nystatin in decreasing fungal viability and reducing the rate of lesion recurrence. Using alternative sources can lead to access to novel therapeutic agents with fewer side effects without the risk of antifungal resistance (Sandai et al., 2016; Torabi et al., 2022). DNase may be an adjuvant for biofilm treatments since it degrades eDNA in the extracellular matrix of biofilms (Rajendran et al., 2014; Hamblin and Abrahamse, 2019). Therefore, we suggest that DNase enabled PDZ diffusion in the extracellular matrix of biofilms, potentiating the effects of PDZ-aPDT and promoting the inactivation of biofilms *in vivo*.

The enzyme associated with PDZ-aPDT reduced the fungal viability of CaS by around 4.26 and 1.97 log_10_, respectively, immediately and 7 days after the treatments. In addition, there was a remission in the oral lesions, around 98.92 and 83.31%, respectively, after 24 h and 7 days. *In vivo* investigation (Carmello et al., 2015) demonstrated that five applications of PDZ-aPDT or antifungal nystatin promoted reductions in the cell viability of *C. albicans* (ATCC 90028) of 3 and 3.22 logs_10_, respectively 24 h after the treatment (Carmello et al., 2016). After 7 days of treatment, a reduction of ~2 log_10_ for PDZ-aPDT and nystatin groups was observed (Carmello et al., 2016). Compared to antifungal, the lack of development of antimicrobial resistance increases further studies using aPDT (Hamblin and Abrahamse, 2019). Thus, our findings revealed that DNase improves aPDT outcomes, being a promising alternative for fungal inactivation, including resistant microorganisms.

DNase I (5 min) combined with PDZ-aPDT decreased the fungal viability in ~2.16 log_10_ and the levels of eDNA in the ECM of biofilms formed by fluconazole-susceptible *C. albicans* (ATCC 90028) (Panariello et al., 2019). When fluconazole-resistant *C. albicans* biofilms were treated with DNase prior to the PDZ-aPDT, there was a decrease of ~1.92 log_10_ in the fungal viability, water-soluble polysaccharides (36.3%), and eDNA (72.3%) (Abreu-Pereira et al., 2022). Here, the fluorescence microscopy images of histological tongue sections 24 h after treatment demonstrated that DNase I before PDZ-aPDT promoted a reduction in fungal polysaccharides, similar to healthy animals (NIC). After 7 days of the treatment, the presence of polysaccharides was observed only in mice infected by fluconazole-resistant *C. albicans*. Still, the fungal viable count was smaller than that in the PDZ-aPDT group. Thus, DNase treatment reduced the fungal polysaccharides *in vitro* (Panariello et al., 2019; Abreu-Pereira et al., 2022) and in the animal model here. Among the components present in the ECM, eDNA, associated with β-glucans and β-mannans, contributes to the organizational integrity of biofilms and antifungal tolerance of *C. albicans* (Martins et al., 2012; Rajendran et al., 2014; Mitchell et al., 2016). Some reports found an association between eDNA levels and increased microbial resistance to antibiotics (Rice et al., 2007; da Silva et al., 2008). Therefore, disrupting the ECM and reducing eDNA levels of *C. albicans* biofilms are essential to optimize antifungal therapies.

The histological sections of the tongues recovered after the treatment with DNase before PDZ-aPDT for both strains (CaS and CaR) demonstrated histological characteristics similar to those of the NIC group (healthy animals). The tissues presented a reduced amount of hyphae/pseudohyphae/blastopore on the keratin layer, minor inflammation in the subjacent connective tissue, and intact muscle tissue. In a previous study, mice treated with 5 applications of nystatin associated with PDZ-aPDT presented normal histological characteristics, which are outcomes very similar to those observed in the present study (Hidalgo et al., 2019). In addition, the inoculation of mice with fluconazole-resistant *C. albicans* promoted an intense inflammatory response in the subjacent connective tissue (Alves et al., 2018; Hidalgo et al., 2019) and 5 applications of PDZ-aPDT decreased an inflammatory reaction from intense to mild (Hidalgo et al., 2019). Here, the control groups (P-L-, P + L-, P-L+, and DNase) 24 h after the treatment presented a large area of hyphae/pseudohyphaes covering the epithelial tissue, which demonstrated acanthosis associated with the papillae destruction.

Moreover, there is an intense inflammation of the epithelium with dilated blood vessels. Seven days after the treatments, the muscle fibers were partially degraded in the superficial region of the tissue in the control groups (P-L-, P + L-, and P-L+ groups). In the sections from animals inoculated with CaR, 7 days after the end of treatment, there were hyphae and pseudohyphae on the keratin layer, suggesting recurrence of the oral infection in the DNase+P + L+ group, but its keratin layer was smaller than that observed for P + L+ group. Furthermore, in the PDZ-aPDT group, there was a return in the oral lesions. Our results demonstrated that DNase before PDZ-aPDT reduces the lesion recurrence rate after 7 days compared to other treatments for fluconazole-resistant *C. albicans*. Therefore, DNase I affected the biofilm composition and hampered the new formation of *C. albicans* biofilm, probably because the complete epithelial restructuring was promoted in the groups treated with DNase+P + L+.

The overall increased occurrences of pathogens resistant to conventional antifungals and the toxicity of drugs have motivated searches for strategies to inactivate fungal species. In addition, the ECM of *C. albicans* biofilms limits the penetration of antimicrobials, antiseptics, and photosensitizers, influencing the efficacy of aPDT and other fungal therapies. In summary, DNase before PDZ-aPDT promoted expressive outcomes by reducing fungal viability and healing the oral lesions in mice. Therefore, this study further demonstrates that DNase is a promising alternative adjuvant for fungal photoinactivation *in vivo* of antifungal-susceptible and -resistant strains.

## Data availability statement

The original contributions presented in the study are included in the article/supplementary material, further inquiries can be directed to the corresponding author.

## Ethics statement

The animal studies were approved by Animal Ethics Committee of the School of Dentistry of Araraquara, UNESP (Case number: 09/2020). The studies were conducted in accordance with the local legislation and institutional requirements.

## Author contributions

CJ: Conceptualization, Data curation, Formal analysis, Funding acquisition, Investigation, Methodology, Validation, Visualization, Writing – original draft, Writing – review & editing. MK: Conceptualization, Validation, Writing – review & editing. PB: Methodology, Writing – review & editing. EM: Writing – review & editing. TS: Methodology, Writing – review & editing. TF: Formal analysis, Methodology, Writing – review & editing. AP: Conceptualization, Data curation, Formal analysis, Funding acquisition, Investigation, Project administration, Resources, Visualization, Writing – original draft, Writing – review & editing.

## Footnotes

^1^https://imagej.nih.gov/ij/docs/install/windows.html
